# *I will write about*: Investigating multiword expressions in prospective students’ argumentative writing

**DOI:** 10.1371/journal.pone.0242843

**Published:** 2020-12-03

**Authors:** Daehyeon Nam, Kwanghyun Park

**Affiliations:** 1 School of Liberal Arts, Ulsan National Institute of Science and Technology, Ulsan, Korea; 2 Department of English Language and Literature, Myongji University, Seoul, Korea; Public Library of Science, UNITED KINGDOM

## Abstract

Multiword expressions are a contiguous series of words in a text. This study examines the phraseological profile based on multiword expressions in argumentative writings in a 120,000-word collection of nonnative prospective university students’ writing. The profile is compared with two sets of American university students’ writing from two corpora that comprise upper-level American university students’ course papers and argumentative essay texts. The data are investigated both quantitatively and qualitatively in terms of the structure (i.e., noun, verb, and prepositional phrases) and function (i.e., stance, referential, and text organizer). The results show some noticeable differences among these sets of writing. The Korean student writers heavily relied on verb phrase-based expressions (e.g., *are a lot of*) in their writing whereas the American students preferred noun phrases. Functionally, the Korean writers underused referential function expressions (e.g., *the idea of the*) compared to their counterparts. In addition, the prospective Korean university students’ writing was found to represent the widest range of multiword expressions whereas the American students’ argumentative course papers exhibited the smallest range. The findings suggest that prospective Korean university students’ writing tends to use more features of verbal conversation while American university students’ writing exhibits features of structured argumentative writing. The implications for teaching writing and limitations of the study are discussed.

## Introduction

Multiword expressions (MWEs) are a contiguous series of words in a text. They are also referred to as multi-word constructions [1], phrases, units, sequences, or bundles [2]. In academic writing, MWEs are considered an indicator of a writer’s competence; thus, they have received much attention. Due to the sheer number of units, computational techniques and resources are commonly used to examine these units. Corpora, or the electronic collection of linguistic data, have been indispensable in researching a large number of MWEs.

Whereas corpora have generally been exploited to identify and analyze patterns of a language in a large-scale study of diachronic and synchronic patterns [3–5], a number of recent studies of corpus linguistics have explored corpora to understand specifically how the multiword sequences are structured and served in different registers and genres (e.g., see [4] for an exhaustive study of spoken and written registers; [6] for university registers; [7] for different academic disciplines). Expanding the perspective beyond studies of individual words, the findings of multiword sequence studies have provided a more systematic explanation of how language users employ these lexical units.

Acknowledging the potential educational benefits provided by the corpus studies, English as a second or foreign language researchers and practitioners have examined the pedagogical use of learner corpora [8]. Such studies have suggested that learner corpora have made a positive contribution to L2 acquisition research [9]. In addition, in English for academic purposes in higher education, corpus-based research has focused on the analysis of L2 writers’ production within academic disciplines.

Although an extensive body of studies has examined students who have already entered the university, little research has been conducted on prospective students’ writing, especially among science and engineering majors. Prospective students’ writing proficiency needs to be examined to reveal their needs and inform the instruction design through investigations of individual words as well as multiword expressions. In an effort to understand the MWEs used by this specific group of learners, the current study aims to analyze the characteristics of their writing through an examination of MWEs. Drawing on the corpus-based studies, this study analyzes their MWEs by focusing on structure and function, the two fundamental dimensions of writing.

Although early studies of MWEs focusing on habitual occurrences of a word with certain words regularly detected the native speaker’s intuition and shared knowledge in a language community [10], researchers have subsequently objected to this approach, arguing that intuition is only useful for obtaining insights into the study of the language [5]. Therefore, the methodological development of corpus linguistic analysis has been widely applied to identify and analyze such recurrent word combinations of a language. The important change of linguistic analysis has allowed linguists to detect, define, and classify not only two-word collocation patterns, but also word combinations with three-, four-, or longer word sequences.

MWE studies have been conducted under various terms, such as *lexical bundle* [2], *cluster* [11], *lexical phrase* [12], *formulaic sequence* [13], *phraseology* [14, 15], *clusters* [11], and *chunks* [16], depending on how they are defined and identified. Unlike many of the earlier MWE studies, the current research is largely corpus-driven. A previous study [17] identified two types of corpus studies: corpus-based research, which describes the language use categories under existing linguistic theory, and corpus-driven research, which establishes new linguistic concepts based on data processing. In using corpora to examine MWEs, Biber [18] exemplified the corpus-driven approach in his lexical bundle research, in which he identified frequent recurrent word sequences in certain registers or genres and, based on the findings, proposed that bundles may not be either grammatically complete or idiomatically grounded. As bundles are simple sequences of words, they can be extracted computationally without being restricted by grammatical or idiomatic structure.

An earlier study [2] identified, described, and categorized the structure and function of lexical bundles in a given genre or register. The structure included three large categories: noun phrase (NP)-based (e.g., *the nature of the*), prepositional phrase (PP)-based (e.g., *as a result of*), and verb phrase (VP)-based (e.g., *has a number of*) expressions. The idea of functional categorization emerged in subsequent studies of multiword structures. While reporting multiword structures in different registers, researchers [4] also discussed the functions of existence (e.g., *the existence of the*), abstract quality (e.g., *the nature of the*), and stance (e.g., *it is possible to*). This taxonomy was subsequently developed further, leading to the introduction of a more sophisticated functional description [19].

Each functional category includes several specific subcategories. Stance expressions serve as a frame for delivering the author’s position; these include epistemic stance (e.g., *it is true that*, *I think that the*), desire (e.g., *if you want to*, *people want to go*), obligation/directiveness (e.g., *the most important thing*, *we don’t have to*), intention/prediction (e.g., *I would like to*, *to be effective in*), and ability (e.g., *not be able to*, *it is impossible to*). Text-organizing expressions are used to build the structure and the relationship of texts serving the function of topic introduction (e.g., *first of all*, *I want to say*), topic elaboration/clarification (e.g., *on the other hand*, *that is to say*), and identification (e.g., *is one of the*, *is the best way*). Referential expressions carry out the function of attribute specification in quantification (e.g., *has a lot of*, *for the rest of*) and tangible/intangible framing (e.g., *has a lot of*, *for the rest of*), imprecision (e.g., *some people argue that*, *some might argue that*), and time/place/text deictic references (e.g., *for a long time*, *when I was a*).

These structural and functional descriptions can be used to explore L2 learners’ use of lexical bundles, especially in terms of how they use the bundles compared to native speakers’ production. This line of study is important for analyzing L2 writing, as it reveals how L2 learners use MWEs in developing writing competence. A number of studies have suggested a structural and functional discrepancy of MWEs between L2 writing and native speakers’ writing [20–22] and have attempted to uncover the underuse and overuse of MWEs [23]. Yet most of these studies have focused on university students, while few studies have examined the writing by prospective students who have not yet received tertiary-level writing instruction. These students’ writing may be different from university students’ writing in full-length essays and course papers. The prospective students would first encounter argumentative writing as they learn to write formal academic essays and would eventually develop their writing skills toward class paper writings.

Drawing upon the previous research, the present study employs three types of corpora to investigate the use of recurrent MWEs in L2: one Korean native speakers’ corpus with argumentative essays written by prospective students and two corpora compiled in American universities—namely, (a) students’ argumentative essays and (b) students’ course papers. Both quantitative and qualitative analyses are undertaken to reveal the differences and similarities among the three types of writing. As for the terminology, MWEs, or simply “expressions,” are used to refer to the multiword sequences.

## Method

### Data

This study compares the argumentative essays produced by nonnative learners of English with the essays collected from American universities. The data comprise three sets: argumentative essays collected from a university in South Korea (hereafter, UNIARG) and argumentative subsets from two existing corpora—namely, the Louvain Corpus of Native English Essays (LOCNESS) and the Michigan Corpus of Upper-level Student Papers (MICUSP). LOCNESS is a corpus of essays and A-level papers from American and British university students. MICUSP is a corpus of advanced student writing, including essays, reports, and response papers collected from courses in a large American university.

We carefully selected these two native corpora to compare with UNIARG, produced by Korean students, for several reasons. First, both corpora consist of comparable genres, as they are a collection of students’ writing in an academic context. Second, we excluded essays by British pupils from the LOCNESS to avoid a possible variability between American English and British English. Third, to make these corpora comparable in terms of lengths, we normalized word frequency first and used an appropriate statistical procedure (i.e., chi-square for non-parametric data). Although this treatment did not remove the problem completely, we believe it minimized the issue.

The nonnative dataset, UNIARG, consists of 746 English argumentative essays with 121,638 tokens. These papers were written by 262 male and 111 female students, a total of 373 prospective students, of English-mediated science and engineering courses in a university in South Korea. The students’ native language is Korean, and the average ages of the contributing male and female students are 19.9 and 20.0, respectively. In Korea, secondary-level students follow a common English education curriculum set by the Ministry of Education; thus, regardless of their college majors, the students receive English education through the curriculum guided by the Ministry. As the university that the UNIARG contributors attend is an English-mediated institution, it is essential for the university to determine students’ English proficiency levels so that it can prepare a customized college English curriculum for the students. Therefore, before their matriculation, the students were required to take academic English proficiency tests, which included an argumentative writing test. The test’s writing task consists of two parts: one responding to a short passage and a lecture video related to the passage (writing time: 20 minutes) and the other responding to a single-sentence prompt (e.g., *Should wildlife be preserved at the expense of human welfare*?; writing time: 30 minutes). The average score of the argumentative writing is 12.5 (SD 5.0) on the TOEFL writing score system. The Institutional Review Board review was exempted for this set of data, as the writing had already been collected as a required placement test and was provided for the research purposes at the researchers’ request on condition that personal identifiers (e.g., names) were redacted.

LOCNESS is a corpus of 436 essays in English totaling 324,304 words produced by native speakers (i.e., American and British students) as well as A-level essays by British pupils [24]. To compare the corpus with the nonnative corpus, only argumentative subsets written by American students are used (hereafter, LOCNESS-AM). The other corpus, MICUSP, is a collection of 829 papers (approximately 2.6 million words) collected in a large American university from four subject divisions: humanities and arts, social sciences, biological and health sciences, and physical sciences. These papers were written by students who earned a grade of A on their papers and, thus, were considered examples of advanced writing in the specific academic discipline [25]. From the corpus, only argumentative subsets were used (hereafter, MICUSP-ARG). Table 1 describes the subsets of the three corpora used in this study.

**Table 1:** Argumentative essay subsets from three corpora of academic writing.

Corpus	Sites of collection	Tokens	Mean length	Essays
**UNIARG**	Korean university	121,638	163	746
**LOCNESS-AM**	American universities	132,812	202	656
**MICUSP-ARG**	A large American university	446,317	2,400	186

### Data analysis

As the purpose of this study is to compare MWEs in different sets of data. For data-processing purposes, this study defines MWE as an *n-gram*, or a contiguous sequence of N words. For example, a 4-gram may be *do you want to* and *I don’t know what*. It is a frequency-based, quantitative approach to investigating multiword expressions. In some traditions of register analysis, this unit is referred to as a *lexical bundle*. Based on their research, this study examines 4-grams, among other possible lengths of n-grams, as they are arguably the most frequently examined kinds of bundles.

There are two conditions for a sequence to qualify as a bundle. First, the sequence should occur more than a certain number of times, or beyond the *cut-off frequency*. In this study, the cut-off is set to 25 times per 1 million words. Second, the sequence must be used in more than a minimum number of source texts, or the *range* of texts. This condition is adopted to exclude idiosyncratic sequences, such as n-grams used by only one author but not others [6]. In this study, the minimum range is set to three texts. Using these criteria, MWEs for investigation are identified as summarized in Table 2.

**Table 2:** Number of MWEs in current study.

Data	Number of MWE tokens	Number of MWE types
**UNIARG**	2254	465
**LOCNESS-AM**	1694	129
**MICUSP-ARG**	1048	44

In analyzing these MWEs, it is important to annotate the essays with part-of-speech symbols, or *tags*. This process is called part-of-speech tagging or grammatical tagging. In this study, texts are tagged using a specialized software program, CoreNLP, developed at Stanford University [26]. MWEs are then obtained by breaking the texts in each document by every instance of the four-word sequence using AntConc, a freely available concordancing program [27], and partially automating the writing simple scripts in the Python computer language (https://www.python.org).

## Results and discussion

This study aimed to compare and contrast the use of MWEs produced by nonnative speakers and students in American universities. After the automatic extraction and manual inspection of MWEs from the corpora, the proportions of the structure and discourse function categories were compared for the MWE types, and chi-square tests were then conducted to confirm how the distribution of the MWEs could be characterized. The standardized residuals (*R*) were also examined to determine which MWE categories were major contributors to the MWE usage differences among the three datasets. As negligible differences were found between the type and token distribution, for the convenience of the presentation, the study only reports the results of the MWE type analyses.

### MWE structure

**Fig 1:**
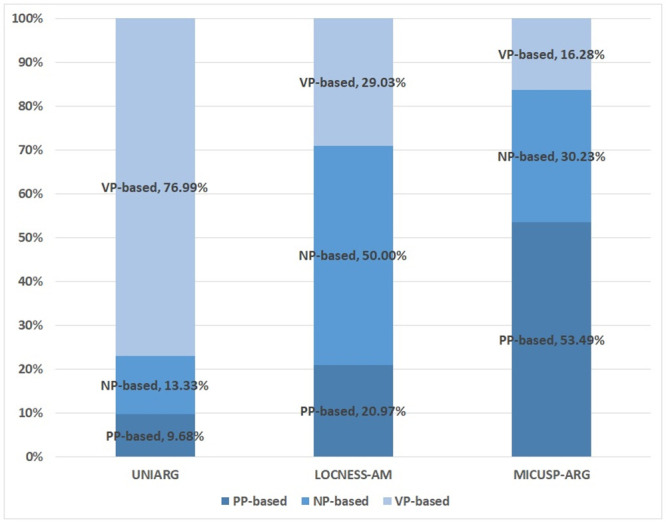
Structural distribution of multiword expressions in three corpora of student writing (types).

As shown in Fig 1, the VP-based MWE in UNIARG covers the dominant proportion (77%) while NP-based and PP-based expressions account for fewer than a quarter of all expression types. On the other hand, in LOCNESS-AM and MICUSP-ARG, the PP-based and NP-based expressions account for much more than half of the MWE structures—namely, 71% and 83%, respectively.

Regarding the proportions of NP-based and PP-based expressions in LOCNESS-AM and MICUSP-ARG, LOCNESS-AM contains a greater proportion of NP-based expressions (50%) and a lower proportion of PP-based expressions (21%) whereas MICUSP-ARG accounts for a greater proportion of PP-based expressions (53%) compared to NP-based expressions (30%).

Table 3 shows how each category consists of specific MWEs and their proportional distribution. PP-based expressions start with a preposition with a noun-phrase fragment, such as *for example there is* (Pattern 1). NP-based expressions include a noun phrase plus post-modifier fragment, as in *the role of the* (Pattern 2). VP-based expressions consist of any combination of a verb or a *be*-copula. In addition, the Other expression category is reserved for grouping verb-related expressions that do not fit Patterns (3) through (9), such as *to sum up the* in Pattern (11).

**Table 3:** Proportional distribution of MWEs’ structure type.

Category	Pattern	UNIARG	LOCNESS-AM	MICUSP-ARG	Example
**PP-based**	(1) preposition +NP fragment	9.68% (45)	20.97% (26)	53.49% (23)	*for example there is*
**NP-based**	(2) NP with post-modifier fragment	13.33% (62)	30.00% (62)	30.23% (13)	*the role of the*
**VP-based**	(3) copula *be* + NP / AdjP	21.79% (78)	5.65% (7)	4.65% (2)	*are a lot of*
	(4) VP with active verb	43.58% (156)	6.40% (8)	4.65% (2)	*have to think about*
	(5) anticipatory *it* + VP /AdjP (+ compl-clause)	3.63% (13)	4.84% (6)	4.65% (2)	*it is important to*
	(6) passive verb (+ PP fragment)	0.00% (0)	0.81% (1)	0.00% (0)	*is shown to be*
	(7) (VP+) *that*-clause fragment	3.91% (14)	3.23% (4)	2.33% (1)	*it means that the*
	(8) (verb/adjective +) *to*-clause fragment	1.12% (4)	0.00% (0)	0.00 (0)	*not easy to use*
	(9) personal pronoun + VP + complement	5.31 (19)	0.00% (0)	0.00% (0)	*I agree that the*
	(10) verb with *Wh*-clause	0.56% (2)	0.00% (0)	0.00% (0)	*know what they are*
	(11) Other expressions	19.57% (72)	8.06% (10)	0.00% (0)	*to sum up the*
**Total**		100% (358)	100% (124)	100% (43)	

Numbers in parentheses indicate raw frequency. All examples are from UNIARG.

Table 3 further indicates that there are more VP-based expressions in UNIARG than the other types of MWE while there are relatively fewer PP-based and NP-based expressions. Specifically, the use of PP-based expressions differs the most among the groups of writing. In fact, of the 45 PP-based expression types used in the UNIARG, nine types start with the fragment *for example*, such as *for example I was*, *for example if we*, and *for example there is*. This is an interesting use of PP-based expressions because it may suggest that Korean English learners’ use of PP-based or NP-based expressions is limited to providing examples after a topic sentence. Therefore, their use of MWEs is more clausal than phrasal, which is contrary to academic writing consisting of a predominant noun phrase with an embedded prepositional phrase fragment [18].

The statistical analysis of the MWE structure types among UNIARG, LOCNESS-AM, and MICUSP-ARG confirmed a significant difference in terms of MWE uses (*χ*2 = 168.584, *df* = 4, *p*<0.01). As presented in Table 4, some of the absolute values of the standardized residual (*R*) are greater than 1.96, indicating a contributing factor of the significant difference (see the numbers in bold). For example, in UNIARG, the PP-based and NP-based expressions were observed less than expected whereas the VP-based expressions were observed more than expected. In this regard, in LOCNESS-AM and MICUSP-ARG, the distribution pattern observed in Fig 1 was statistically confirmed to have different uses of NP- and PP-based expressions.

**Table 4:** Chi-square contingency table: MWE structure type.

Data		PP-based	NP-based	VP-based
**UNIARG**	Observed Count	45	62	358
	Expected Count	69.16	100.80	295.04
	Standardized Residual	**-2.91**	**-3.86**	**3.67**
**LOCNESS-AM**	Observed Count	26	62	36
	Expected Count	18.44	26.88	78.68
	Standardized Residual	1.76	**6.77**	**-4.81**
**MICUSP-ARG**	Observed Count	23	13	7
	Expected Count	6.40	9.32	27.28
	Standardized Residual	**6.57**	1.20	**-3.88**

χ^2^ = 168.584; *df* = 4; *p* = 0.000, Effect size: 0.52 (medium effect size); Cramer’s *V*: 0.365.

### MWE function

**Fig 2:**
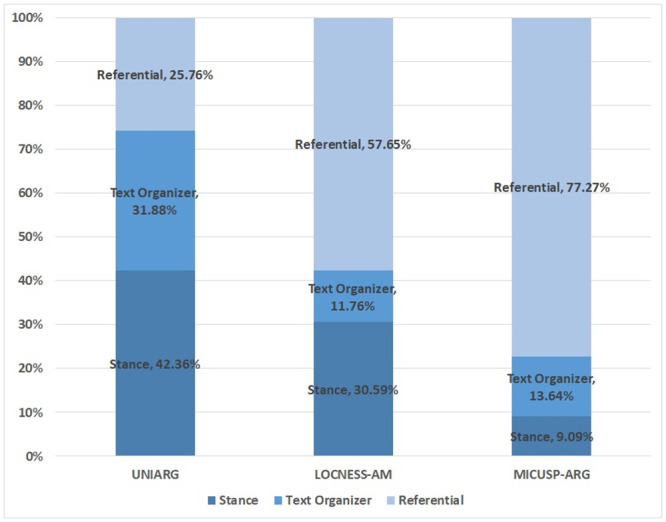
Functional distribution of multiword expressions in three corpora of student writing (types).

Fig 2 presents the distribution of the MWE function categories. A distributional difference emerged between UNIARG and the other native speakers’ corpora.

Unlike the greater proportion of the referential expressions in LOCNESS-AM and MICUSP-ARG, in UNIARG the stance expressions account for the majority of the distribution, followed by the text organizer and referential expressions. An outstanding difference also exists between text organizers and stance expressions. LOCNESS-AM includes more stance expressions than text organizers. However, in MICUSP-ARG, the text organizer is about 9 percentage points higher than the stance MWEs.

The statistical analysis of the lexical bundle function types among UNIARG, LOCNESS-AM, and MICUSP-ARG confirmed that a significant difference exists in terms of the functional uses (*χ*^2^ = 58.699, *df* = 4, *p*<0.01). As presented in Table 5, the referential expressions are highly frequent in LOCNESS-AM and MICUSP-ARG, whereas the Korean learners of English do not seem to rely on this functional expression in their writing.

**Table 5:** Chi-square contingency table: MWE function types.

Data		Stance	Text Organizer	Referential
**UNIARG**	Observed Count	97	73	59
	Expected Count	81.24	56.93	90.83
	Standardized Residual	1.79	**2.13**	**-3.34**
**LOCNESS-AM**	Observed Count	26	10	49
	Expected Count	30.15	21.13	10.94
	Standardized Residual	-0.76	**-2.42**	**2.63**
**MICUSP-ARG**	Observed Count	4	6	34
	Expected Count	15.61	10.94	17.49
	Standardized Residual	**-2.94**	-1.49	**3.96**

χ^2^ = 58.699; *df* = 4; *p* = 0.000, Effect size: 0.40 (medium effect size); Cramer’s *V*: 0.286.

The referential expressions in UNIARG include 22 types of quantity specification expressions, and many of them are based on the phrase *a lot of* (e.g., *a lot of people*, *a lot of time*, *have a lot of*, *with a lot of*):

- First, technology have a lot of possibility. Developed technology can making things do any function. So children study the…. (UNIARG)
- Of course, they have few negative effects. But, it can release with a lot of positive effects. For example, we can consider about health problem. (UNIARG)

On the other hand, quantity specification expressions are rarely used in either LOCNESS-AM or MICUSP-ARG. For example, in MICUSP-ARG, *a great deal of* is one of the two types of quantity specification expressions, with the other being *the rest of the*. Similarly, in LOCNESS-AM, although there are more types of quantity specification expressions than in MICUSP-ARG, the number of types is still considerably smaller than UNIARG (e.g., *a large number of*, *most of the time*, *the majority of the*):

- Party identification accounts for a great deal of the logic that determine voting behavior. (MICUSP-ARG)
- The roots of the problems in higher education lie with Napoleon’s policy on education which was to educate a well-trained small elite to run the country, while guaranteeing that the country remained stable by ensuring that the majority of the population was not over-educated. (LOCNESS-AM)

In fact, the more frequent referential expressions in LOCNESS-AM and MICUSP-ARG are intangible framing attribute expressions rather than quantity specification expressions:

- As mentioned previously, one of the main claims of the advocates of capital punishment focuses on the idea of the death penalty acting as a deterrent. (LOCNESS-AM)
- The arguments for and against EC and the maintenance of its current status of limited availability provide an example of the way in which personal female decisions become political. (MICUSP-ARG)

An exhaustive study of the Korean English learners’ writing [28] found that indefinite quantifiers such as *all*, *many*, *a number of*, and *a good/great deal of* can function as hedges. However, from the quantity-specifying expressions in UNIARG, these expressions may serve a different function than managing the writer’s degree of certainty. The specific examples of referential expressions may show that the Korean writers would want to use the quantity specification expressions to justify their argument—that is, the writers simply want to justify their argument by majority (e.g., if 10,000 people think X is right, then X is right). This kind of argument is not constructed on sound logic or firm evidence, but rather the unspecified many. This is clear and obvious because of the observation that the relationship between the sentence with the quantity-specifying expressions and the subsequent sentence is the premise and the specific example, respectively. However, in the native and advanced writers’ writing, the authors attempt to identify abstract characteristics or particular attributes of an entity as especially important. The use of tangible or intangible/intangible framing attribute expressions could be an indirect, but more powerful way to deliver the writers’ arguments.

Although no distinct contrast emerged in the text organizer expression uses between UNIARG and the other datasets, it seems that the Korean writers relied on text-organizing expressions more than the native writers. Furthermore, the predominant text-organizing expressions were topic introduction/focus expressions whereas native writers used other expressions, such as topic elaboration/clarification or reasoning expressions:

- So I will write about how technology influence children I think technology has made children more creative. There are two reason in my opinion. (UNIARG)
- On the other hand, maybe it’s simply due to the fact that the crucial task of raising children has no dollar value attached to it, and is therefore symbolically worthless in our market-driven culture. (LOCNESS-AM)
- If this is all our ideas of efficacy, necessity, and productive power amount to, it is clear that we could have no impressions sufficiently resembling this idea in order to warrant our trust in it. (MICUSP-ARG)

Furthermore, the functional MWE usage difference between L1 and more proficient writers’ writing becomes evident in the use of obligation/directive expressions in the stance category. Although the stance category in UNIARG and LOCNESS-AM is not a major contributor characterizing the difference, a close investigation of subcategories would provide clues for the distinction. In UNIARG, of the 97 types of stance expressions, 27 types (28%) are obligation/directive expressions while the same subcategory accounts for only 15% of the stance expressions in LOCNESS-AM (e.g., *it is important to*, *of the most important*, *it should not be*, *should not be allowed*). Moreover, only one obligation/directive expression is found in MICUSP-ARG (e.g., *it is important to*).

- At last, in recently (*sic*), young people’s crimes are increasing. So we have to do something that stop making problems. (UNIARG)
- Second, about the pollution. The passage says that the internal-combustion engine is making too much pollution, so we need to change them into hydrogen-based engine cars. (UNIARG)
- Another reason why it should not be banned is because foxes give chickens a cruel death when they kill them. (LOCNESS-AM)
- Clomiphene citrate is no longer used in IVF but it is important to realize the effects of the drugs were not seen until nearly 20 years after the drug was first administered in women. (MICUSP-ARG)

A close investigation of how obligation/directive expressions are used in stance expressions would contribute to an informative explanation to understand their writings. As shown in the preceding examples, Korean learners of English tend to rely on VP-based expressions. In fact, this is an interesting use of stance expressions as previous researchers [4] classified the obligation modal verbs across the registers of conversation, fiction, news, and academic prose. In their classification, the modal *have to* is used 4.5 times more frequently in conversation than in academic prose. Therefore, Korean writers’ use of stance expression shows a conversation register pattern in their argumentative writing. Another finding is that L1 writers typically use the structural type anticipatory *it* + VP/adjective in relation to the obligation/directive function, but this connection is rarely found in Korean writers’ writing. Likewise, L1 writers typically seem to use the extraposed MWEs with verb predicates in a passive construction. This type of structure is known to be frequent in academic prose in reporting the stance of the writer [4]. In contrast, the structure is not as common in Korean writers’ essays.

The structure and functions of MWEs by Korean students are summarized as follows. First, there is a significant difference in the structure of MWEs between the nonnative students’ writing and the American students’ writing. The difference is most remarkable in the number of noun and verb phrases. Although the great majority of MWEs in the nonnative students’ writing is VP-based expressions, these expressions only represent a much smaller proportion of data in the American students’ essays and course papers. In stark contrast, NP-based MWEs are much more frequent in the American students’ writing than in the Korean students’ writing. The difference implies the American students’ preference for NP-based expressions and Korean students’ preference for VP-based expression in academic writing.

The differences seem to reflect the developmental status of English in nonnative writing. In the written register, complex thoughts are expressed in nominal forms, as they condense arguments into noun phrases to further develop the arguments [29, 30]. Register studies based on large corpora have observed this tendency, finding nominalization to be a characteristic of the written register [31, 32]. Similarly, when learning English, the development of writing skills is reflected in the gradual shift from verbal to nominal forms [33, 34].

Second, the significant difference found between the Americans’ and Koreans’ writing relates to frequent discourse functions. American students’ writing consistently shows more referential expressions on the one hand and fewer stance expressions on the other hand. Given the characteristic of the argumentative genre, it is not surprising that a number of stance expressions are used (e.g., *I think it was*, *the fact that the*). Writers use stance expressions to render their voice persuasive. However, in nonnative students’ writing, there is a clear overuse of the stance function compared to native students’ writing. Again, in contrast with the stance function, there is a strong preference for the referential function in the American students’ writing in the course papers, followed by American students’ essays and the nonnative students’ essays.

An integrated interpretation of this contrast is that the American students use conceptual devices (e.g., categorization, comparison and contrast, presentation of ideas in a logical progression), which are the functions that the referential expressions perform. These devices allow the writers to base their arguments on information and factual evidence (e.g., *on the basis of*, *as a result of*), rather than personal stance (*I strongly argue that*, *I don’t know what*). In terms of the stance and referential expressions, discourse organizers are found to operate in a similar way.

When comparing native and nonnative students’ writing, one sub-function—topic introduction—is significantly more frequent in the latter than in the former. In contrast, the topic elaboration sub-function occurs more frequently in native students’ writing. Thus, it can be inferred that, in nonnative students’ writing, topics are often introduced with a stance expression, but they are not fully elaborated with supporting evidence and information, which are often provided in referential expressions. The exact opposite is true in native students’ writing: Once the native students have introduced the topic, they elaborate on it with information and textual evidence in referential expressions. In addition, in the referential expressions, the quantity specification used in the writing of the Korean learners of English would serve another function that has been in explained in the literature—namely, in their argumentative writing, L2 writers may want to make their argument stronger by relying on the “argument by majority.”

The findings in this study should be interpreted cautiously in terms of the comparability of data. First, Korean students’ writing may be influenced by their L1, the Korean language, and its rhetorical style. However, the data in this study do not allow us to track L1 influence and writing style on their use of multiword expressions. Second, it was not possible to compare the prospective Korean students’ essays with those produced by advanced Korean students. Third, the three corpora were compiled in different settings; data in UNIARG and LOCNESS were collected in exam settings, while the data in the MICUSP were collected as homework assignments.

## Conclusion

The present study compared MWEs in academic English in Korean students’ essays, American students’ essays, and upper-level course papers of American students. Although all of these texts are argumentative by genre, there emerged noticeable differences among the three author groups. The findings from this study offer some insights into the structural and functional description of academic English, a special variety of English, yet they should be interpreted with some limitations in mind. First, argumentative writing does not represent the variety as a whole, despite being a very common academic written genre. This limitation can be overcome with larger corpora balanced with a wider array of genres (e.g., exposition, analysis, lab reports). Second, due to the technical limitations of this study, it was not possible to identify all MWEs accurately. Although the researchers carefully cross-examined the data in order to resolve any discrepancies, it was difficult to maintain high accuracy at all times throughout the entire process. This challenge can be addressed in a future study with the support of a computer program that automates the process. Third, due to the lack of resources, we used two native corpora, LOCNESS and MICUSP. However, these students are university students, while the Korean students are prospective students. For a more accurate comparison, there should be corpora of native prospective students. Finally, there is a confounding factor (e.g., language proficiency) operating behind the scenes. As the Korean students’ proficiency ranged from beginner to low intermediate, the findings in this study do not represent English used by advanced learners. Future studies should investigate the differences in MWEs across proficiency levels or novice and professional academic writing to uncover the nature of L2 writing proficiency development in an academic context.
